# Research on dementia and inequalities in care and support – a map of the terrain and discussion of key issues

**DOI:** 10.1186/s12939-025-02601-4

**Published:** 2026-04-01

**Authors:** Michael Clark, Louis Compton, Nazak Salehi, Martin Knapp, Adelina Comas-Herrera

**Affiliations:** Care Policy & Evaluation Centre, London School of Economics & Political Science, London, UK

**Keywords:** Dementia, Inequalities, Inequity, Intersectionality, Protected characteristics

## Abstract

**Background:**

Inequalities and inequities in health and social care are a significant focus of attention. We need to understand these issues in dementia care as well, particularly given the projections for increases in the number of people being affected by the condition and the related rise in costs to individuals, families, communities and nations. We provide a map of the terrain of research concerning inequalities in dementia diagnosis, care and support in the United Kingdom (UK), and discussion of key issues.

**Methods:**

We map key literature related to our objective, particularly regarding the characteristics of people protected in law in the UK, plus socioeconomic status and geography (urban/rural) to map contours of the evidence and key issues. We focus on evidence from the UK, but have also drawn on international evidence as insightful for the map.

**Results:**

The terrain of inequalities in dementia care is complex. Inequality has not tended to be an explicit lens through which researchers have sought to understand dementia care. Literature aligned to the issue chiefly focused on a single characteristic of people, such as ethnicity. Consideration of interactions of people’s characteristics and experiences—an intersectional approach – has not been a significant focus. Consideration has mainly focused on the barriers people face, e.g. obtaining a timely diagnosis and access to appropriate support, with less robust evidence about means of removing these.

**Conclusion:**

More should be done to understand the systemic barriers creating inequalities in accessing dementia diagnoses, care and support, particularly understanding the related intersections of people’s characteristics and experiences, and how to remove such impediments to accessing high-quality support. Recommendations for research to build a robust evidence-base to help improve dementia policy and planning to address systemic inequalities are provided, including that a stronger ‘inequalities’ lens is used and one that draws on an intersectional understanding of people.

## Introduction

Inequalities confronting people in health and social care is a longstanding concern in England (e.g. [1, 2]), and is of heightened policy concern since the Covid-19 pandemic and with ongoing economic pressures (e.g. [3–5]. Indeed, these inequalities appear to be not only enduring but also increasing. Attention has also begun to focus on inequalities in dementia care and developing a more detailed and sophisticated understanding of how they operate, what impacts they have and how they might be addressed (e.g. [6–8]).

Globally, dementia is a high priority, recognising rising demands on individuals, families and societies, and inequalities in care within and between countries [9, 10]. United Kingdom (UK) estimates are for the number of people living with dementia to increase, from 650,000 in 2015 to 900,000 in 2025, 1.35 million in 2040 [11], and potentially 1.60 million by 2050 [10]. This will mean associated increases in the costs of care,for example, projections are for UK expenditure on dementia support to rise by 249%; from £23.0 billion in 2015 to £80.1 billion by 2040 [11].

The *Prime Minister’s challenge on Dementia 2020* [12]:7), a strategic vision for dementia care in England, stated:

our vision is to create a society by 2020 where every person with dementia, and their carers and families, from all backgrounds, walks of life and in all parts of the country – people of different ages, gender, sexual orientation, ability or ethnicity for example, receive high quality, compassionate care from diagnosis through to end of life care.

Yet, whilst acknowledging evidence that people from some communities face additional barriers to accessing support, no further analysis of the implications of such inequalities was evident.

More recently, proposals for a *Major Conditions Strategy* [13], encompassing cancers, cardiovascular disease, musculoskeletal disorders, mental ill health, dementia, and chronic respiratory disease, identified a need to systematically understand and address inequalities in care for these conditions. The dementia section, however, again contained no detailed analysis of inequalities, with the tone being one of a need for better evidence.

This gap in our understanding of inequalities has impacts on improving practice as well as for policy development. For example, whilst commissioners of dementia services across England recognise inequalities facing people from some ethnic minority communities, few localities report services specifically aimed at addressing the issues [14]. To stimulate and help focus work addressing inequalities in dementia care we map key evidence and issues related to inequalities in dementia care. This is part of a larger research programme (STRiDE England) aimed at improving dementia care policy and practice. We have, then, focused on evidence from the UK, but added more international evidence to the map as it provided insightful, relevant evidence to that country.

### Understanding the landscape of dementia and inequalities

Our focus is on mapping the broad contours of the evidence on inequalities in dementia care and support for people to understand the state of knowledge and key issues, beginning to bring together a dispersed evidence base. In doing this we are contributing to an emerging better understanding of these issues (e.g. [7, 8]).

The literature considering inequalities in general and as a specific key focus in relation to dementia is limited. More has focused on specific groups of people with shared characteristics. Hence, we structured our search strategy around specific characteristics that might be linked to experiences of inequality. These characteristics include those protected under the Equality Act 2010, plus the impacts of socioeconomic status and geography. Extracted data from pertinent literature were synthesized narratively for each focus, with findings organized for each characteristic into two sections: obtaining a diagnosis, and accessing care and support. We have not considered inequalities relating to possible prevention of dementia, nor those between dementia and other conditions. After considering a search strategy focused on ‘inequalities’ and finding this to be too specific to highlight important literature we were aware of, we searched databases for literature on ‘dementia’ (and related terms) linked to search terms for each of the characteristics we discuss below. Two authors each led the searches for specific characteristics. Abstracts were then screened for relevance by the two authors with a third reviewing any uncertain articles. The final sets of papers for each characteristic were then examined by the team for any obvious errors of inclusion or omissions of important papers known to us. Within each focal characteristic data were extracted from relevant papers relating to our two concerns of obtaining a diagnosis and accessing care and support. Once the maps for individual characteristics were completed, we reflected on the whole landscape to identify the key overarching themes discussed below.

Inequalities is a complex phenomenon requiring careful analysis when seeking to improve people’s experiences of and outcomes from dementia care, including whether the significant question is inequality (uneven distributions, such as access to resources) or equity (unfair or unjust differences), or combinations of these. Health inequality, for example, refers to a measurable aspect of health that varies across people, whilst health inequity denotes an unjust difference [15]. Further, providing people with more equal quantities of the same thing may not address imbalances in equity. For shorthand in this paper we will continue our discussion referring to ‘inequalities’, but recognising this issue of the importance of inequity and of the need to particularly address unfair differences between people.

We will now discuss each characteristic we examined in turn, identifying the key evidence concerning our two organising themes of *obtaining a diagnosis*, and then *access to dementia care and support*. We start with ethnicity.

#### Ethnicity

##### Obtaining a diagnosis

There is a complex, not fully clear pattern of diagnosis across ethnic groups and, hence, of inequalities, but differences clearly exist in dementia diagnosis between groups (e.g. [16–19]). People from some minority ethnic groups tend to receive diagnosis later in the progression of their dementia and at a younger age with increased severity [16, 17, 19–21]. Mukadam et al. [16] found people from Black and Asian backgrounds have lower cognitive scores at diagnosis, potentially indicating increased severity linked to delayed diagnosis. Similarly, Tsamakis et al. [19] and [21] found that Black African and Black Caribbean people present with dementia at a later stage. [22, 23] suggest minority ethnic groups report a longer duration of memory loss than non-minority ethnic groups at referral to dementia services.

UK Asian ethnic groups are reported as less likely to receive a dementia diagnosis compared to the white majority population, reflecting underdiagnosis or lower incidence of dementia [18]. In the same study, UK Black ethnic groups were more likely to receive a dementia diagnosis compared to the white majority population. These researchers suggest this only partly reflects their higher dementia incidence and thus underestimates the higher risk of dementia in Black ethnic groups.

##### Access to dementia care and support

People living with dementia from minority ethnic groups often have different experiences with access to and use of dementia services compared to majority communities (e.g. [19, 24–30]). Further, dementia inequalities by ethnic groups come against a backdrop of other health inequalities including increased prevalence of long-term conditions and multimorbidity (e.g. [30]). South Asian and Black people living with dementia are reported to die younger compared to non-minority ethnicity groups with dementia [17].

Prescription practices highlight ethnic inequalities, with Black African and Black Caribbean groups less likely to be prescribed an antidepressant and polypharmacy being more common among all ethnic minority groups [19]. Minority ethnic groups are reported to receive incorrect medication for dementia treatment at higher rates and, when prescribed correct medication, it is often for a shorter duration [19, 22, 27]. [27, 31] found Asian people to be less likely prescribed anti-dementia drugs when they were potentially indicated. Socioeconomic status should also be considered alongside medication findings by ethnicity [19].

Lack of knowledge of dementia, stigma, and/or viewing care as a familial responsibility are barriers to accessing support for some ethnic minority groups [24, 26, 28, 29, 32]. Delayed help-seeking is common, with some communities viewing memory problems as an inevitable part of normal ageing to be lived with [33]. In addition, concerns about being seen as less a person may also be a barrier for accessing support for some minority ethnic groups [25]. Limited awareness of dementia and local services, linguistic and cultural communication barriers, and historical medical racism or previous negative experiences with healthcare also hinder access [7, 24]. These issues should not, though, place the burden of addressing inequalities within the communities themselves. Rather, system-wide and community-engaged approaches to helping individuals and communities should be adopted.

#### Gender

##### Obtaining a diagnosis

We examined inequalities in dementia by sex (biological category) or gender (a wider cultural/social category). We use ‘gender’ as our preferred term for this discussion.

Gender-based differences have been found in access to dementia diagnosis [34–36]. A finding is of women often being diagnosed later in their dementia prognosis, partly connected to them living longer than men on average and so are more likely to live alone and harder for services to identify [35, 36]. Another factor in later diagnosis may be women performing better in diagnostic tests, leading to delayed diagnosis and more rapid decline after diagnosis [35]. Barriers to men receiving a timely diagnosis can be lower levels of seeking medical attention and stigma surrounding dementia [34].

##### Access to care and support

Women disproportionately bear the burden of caring for family members with dementia, often facing additional caregiving expectations despite being at higher risk of developing dementia themselves [37] [35, 38, 39]). Evidence indicates that women provide care for longer periods than men and spend more hours per day on caring, resulting in increased burden and stress for women. Gender inequalities persist in caregiving responsibilities, impacting daughters more heavily than sons and leading women to be more likely to reduce their paid employment hours. Men are often unable/unwilling to cook for wives who develop dementia, or they completely take over cooking and other tasks, potentially indicating men are less likely to facilitate the continuing autonomy of partners living with dementia [38, 40].

Inconsistencies in medication prescribing also relate to gender inequalities [41, 42]. Men living with dementia in the UK are less likely to be taking psychotropic medication than women living with dementia [41]. In Canada anticholinergic medications were more often prescribed to women than men [42].

Women with dementia experience lower rates of use of primary care services and longer periods of hospitalisation [41–43]. In part this may result from persistent gender imbalances in carer roles and the interplay with relative gender longevity, i.e. men are more likely to have wives to look after them.

#### Disability

Reviewing the literature on disability and dementia would be a substantial undertaking. To keep this aerial view manageable, we focus on living with physical and or learning (intellectual) disabilities broadly and interactions with dementia and accessing related support.

##### Obtaining a diagnosis

Several studies have found a more difficult pathway to diagnosis of dementia for many living with disabilities compared with those without them (e.g.) [44, 45]. Communication difficulties have been seen to hinder diagnosis pathways for people living with some disabilities [44–46]. As an example, Young et al. [44] found that deaf people are less likely to be diagnosed with dementia than hearing people.

Symptoms of learning disabilities can overlap with common problems experienced by people with dementia, e.g., memory problems, anxiety, depression, or distress, making it more difficult to distinguish dementia symptoms [46]. In addition, Zeillinger et al. [45] found globally that dementia tools for assessments are not tailored for individuals with learning disabilities, hindering early detection. There are higher rates of dementia in people with some learning disabilities, in part potentially because of unaddressed but modifiable preventative factors, e.g. related to generally poorer access to health care. Delayed dementia diagnosis can also result in negative assessments of people, potentially being labelled as “challenging”, or similar labels.

##### Access to care and support

Some have reported services to be unsuitable for optimal care for people living with dementia and disabilities [46, 47]. Cleary & Doody [46] noted that following diagnosis, caregiving posed difficulties for staff with staff being unable to distinguish between underlying intellectual disability and dementia-related problems. Additionally, [47] found that people living with learning disabilities are less likely to age in-place, i.e. in their usual place of residence and communities, potentially creating poorer experiences if people develop dementia.

#### Sexuality

LGBTQ + (lesbian, gay, bisexual, transgender, queer, and others) communities often underrepresented in health and care research [48], leading to a lack of evidence of their experiences. There may be a need for oversampling of densely LGBTQ + populated areas to address this challenge [49] to develop a better evidence base for more inclusive dementia care.

##### Obtaining a diagnosis

Evidence highlights that LGBTQ + people living with dementia are generally at greater risk of negative health, particularly mental health, and related health inequalities [13, 50]. Community and family stigma and cultural values lead some in LGBTQ + communities to avoid consultation with healthcare professionals, and, because of this, dementia diagnoses are often delayed or not received [48], causing problems in England where formal diagnosis is usually a gateway to receiving necessary and timely treatment, care and support.

##### Access to care and support

A theme throughout the literature is differences between LGBTQ + populations and the general population which can have an impact on support when people are older or unwell. As an example, LGBTQ + populations are less likely to have children, meaning that older LGBTQ + people living with dementia had an increased risk of requiring formal care earlier [51].

Our mapping found that LGBTQ + people are a particularly underserved population, for example how they can feel like an invisible group of people in care settings [48, 52, 53], resulting in access inequalities. Care settings are often frequented by those with dementia, yet throughout the literature care settings were labelled as heteronormative, assuming heterosexuality, and, hence, experienced as discriminatory and making services unrelatable and compromising to LGBTQ + identity [50, 51, 54].

The LGBTQ + community is also underrepresented amongst staff, and social care staff are often perceived as lacking cultural competencies, resulting in poor staff-client relationships and communication [50, 51]. Negative staff attitudes have been seen as barriers to care access [55]. With a reluctance by LGBTQ + people, including those living with dementia, to access care they need when they require it, they become underprepared for later-stage disease progression, making the future more difficult and distressing [50, 51, 54].

#### Age

In considering age and potential inequalities in dementia we primarily focused on young onset dementia because of its distinct challenges and underrepresentation in research and in service provision compared to the general population and within the field of dementiaa. As with other characteristics, different terms have been used to describe this phenomenon, including early onset dementia and young people with dementia, but here we use young onset dementia. We also touch on inequalities in relation to obtaining a diagnosis and accessing care and support for older people with dementia as we recognise the different difficulties they face.

##### Obtaining a diagnosis

People experiencing young onset dementia can experience delayed diagnosis compared to older people developing dementia. This can be for many reasons, including poor clinical understanding of young onset dementia [56, 57], and/or complications from young onset dementia often being a secondary condition to, or masked by, another (e.g. brain tumour, stroke or Parkinson’s disease), combined with a lack of specialist training for clinicians, can add to mis/late diagnosis [58–61]. Limited service capacity can also hamper timely diagnosis [62], as can low public understanding of the condition [31, 57], and there is often a misconception that dementia symptoms (i.e. memory changes) are a normal sign of aging Parker et al. [63] which can impact timely diagnosis, especially for older adults. This misidentification of dementia symptoms in older people is not limited to those experiencing them but also made by healthcare professionals on occasion [58, 59].

##### Access to care and support

Inequality in delayed diagnosis for those with young onset dementia is linked to subsequent weakened support systems for people with young onset dementia as relationships may break down prior to diagnosis [64]. Many experiencing young onset dementia doubt or reject their diagnosis due to their younger age, delaying seeking care [56, 65]. Studies also suggest that people with young onset dementia experience feelings of stigma, fear and embarrassment around their diagnosis leading to further social isolation [66]. Social isolation and loneliness are risk factors for dementia, and older people are particularly vulnerable Lazzari & Rabottini [67]. Those older and socially isolated people living with dementia are also potentially at greater risk of developing more severe behavioural and psychological symptoms of dementia which can make care more challenging Sun, Matsuoka & Narumoto [68].

People with young onset dementia often receive standard dementia care tailored to an older population and not suitable to their stage of life [58, 59, 64, 69]. Services not meeting their social and activity needs often results in a person with young onset dementia feeling purposeless Tan et al. [70]. Also, since the Covid-19 pandemic in England many support services have changed operational arrangements, e.g. moved online, which has led to increased issues regarding support accessibility, especially for those older people living with dementia who do not necessarily have the technological skills to access these services [58, 59].

Addressing different abilities and care needs, and longer use of informal care may increase carer burden in the context of young onset dementia [57, 64, 66]. Carers additionally often must take on new responsibilities and financial pressures when those diagnosed with young onset dementia can no longer continue with their previous family, monetary, and/or occupational commitments [56, 64].

Young onset dementia is associated with other characteristics and inequalities covered by this mapping. Rates of young onset dementia have been found to be higher in ethnic minority groups and was linked to some learning disabilities [65, 71]. At these intersections it is likely that the effects of inequalities are compounded, leaving these individuals additionally marginalized.

#### Socioeconomic status

Socioeconomic status is well evidenced as a social determinant of health inequalities generally (e.g.[1, 72]). Here we discuss the contours of evidence on socioeconomic status and inequalities in dementia.

##### Obtaining a diagnosis

The evidence on diagnosis and socioeconomic inequalities is not clear, reflecting a complex mosaic and evidence gaps. Evidence [8, 73–77], indicates:

- Potential under-diagnosis of dementia in more deprived neighbourhoods, with the situation likely to be complicated by higher levels of other poor health conditions masking dementia diagnosis;
- But also, potentially higher levels of diagnosis in deprived areas, possibly related to comparatively increased contact with primary care and, hence, more opportunities to identify dementia.

Ongoing examination of the complex interactions of deprivation, dementia rates and diagnosis is needed.

##### Access to care and support

The relationships between socioeconomic status and access to care and support are similarly complex to map, particularly given the challenges of factoring in levels of need. Evidence across the literature is inconsistent, emphasising multifaceted interactions between people and their social circumstances, dementia, socioeconomic status and the often-challenging organisation of care systems.

Potentially people with dementia residing in poorer areas face inequalities regarding access to some care, but this may be less clear when allowing for levels of need [58, 59, 74]. Literature suggests that wealthier individuals are admitted to care home earlier in the dementia pathway [58], whilst in terms of home care, there were more wealthy individuals using these services than poorer people [78, 79]. People of lower socioeconomic status in England were found to have consistently lower prescription rates for anti-dementia medications than more wealthy individuals [41]. People living with dementia in the most deprived areas have been found to be at greater risk of emergency hospital admission whilst also having a lower probability of elective admission [76].

With regards to care funding, a phenomenon of the *“middle-class squeeze”* has been observed [58, 59]. The social care funding system in England means that those most deprived (with low levels of savings and assets) receive financial support from the state to fund necessary care, and although the wealthiest do not receive this financial support, they have greater financial capacity to fund their own care. It is those who do not quite qualify for government financial support, and who struggle to sustainably fund their own care, that are potentially left un- or underserved by the system.

Socioeconomic mechanisms of inequalities are closely linked to those of ethnicity and geography [79]. Systemic solutions considering these interactions are needed as an isolated intervention at one characteristic may not address the whole of the web of inequalities.

#### Geography

Geography, the characteristics of the place a person lives in, is another complex domain in understanding dementia inequalities. As such we cannot explore it here its entirety, nor its overlaps with socioeconomic status. The All-Party Parliamentary Group on Dementia [80] recognised the importance of this dimension of inequalities in its report on unwarranted geographical variations in dementia diagnosis rates.

For this aerial overview of knowledge about inequality and dementia we focused on rural/urban aspects of geography. Health and social care have been found to be unequally distributed by area across England [81, 82], with rural areas generally having lower spend on services.

##### Obtaining a diagnosis

Evidence points towards those residing in rural areas having poorer access to diagnosis of dementia [8, 73]. This may relate to lower levels of services, as discussed next.

##### Access to care and support

People living with dementia in rural areas have been seen to be more likely to visit hospital emergency departments, but less likely to be admitted to emergency hospital care, a contradiction possibly due to a lack of local service capacity [82]. Having generally fewer local services in rural areas may mean that people with dementia there rely more on GPs for support and as gatekeepers to services [82, 83]. Without local specialist dementia services, rural residents have been found to be more likely to be seen by a GP alone, and less likely to be referred to a dementia specialist for formal diagnosis or treatment [60, 83].

Access to care was found to be more difficult in rural areas due to increased distances to care, shortages of transport, and difficulties in navigating rural landscapes due to personal physical deterioration and/or cognitive decline, all often contributing to greater social isolation [83–89]. These difficulties are reflected in greater rural care costs, adding to a reluctance to access services when paying for them directly. All of which means there is often a greater carer burden in rural areas [87, 88]. People living with dementia in rural areas are characteristically more likely to be living with relatives, creating a potentially greater rural carer burden and some negative impacts on carer wellbeing [86, 89]. An absence of carer support services and a shortage of dementia trained staff in these areas can contribute to the inequalities in care, [83, 86, 90, 91]

Rural/urban inequalities with relation to dementia care may interact with other inequalities in multiplying negative effects. Further research regarding geographical inequalities in dementia should focus on interactions with socioeconomic status, ethnicity, age, and access to other services [87, 92].

## Discussion

In the foregoing we have provided a map of evidence on inequalities and dementia care. This is a highly complex terrain that has not been well examined as a whole, but rather as a series of isolated parts, such as research on specific minorities. Whilst we have not been able to assess evidence in detail for each topic, the value of this work is providing that overview and then being able to highlight some crosscutting issues. The widespread and complex challenge of inequalities in dementia care is of course a key issue evident from the aerial view. We have noted specific issues of inequalities regarding access to timely diagnosis and post-diagnostic support for each characteristic discussed above.

In this discussion we highlight three key overarching themes for the future of research, policy and practice improvement to address inequalities in dementia care. These are i) the general lack of a strong inequalities lens to understand the issues; ii) the need for an intersectional perspective to understand the causes and impacts of inequalities; and, iii) the need for more evidence about policy and practice interventions to address the identified inequalities. These points are interrelated, but we discuss each in turn. Our focus has been on England and dementia care there, and this remains so in our discussion, but our themes are likely to apply to other parts of the UK and even many other countries.

Our first overarching theme is the observation that *the perspective of inequalities has not been a commonly employed explicit lens in dementia care* for understanding the challenges that many individuals and groups face. Although that perspective is beginning to be used more, the focus in understanding unfair dementia care has tended to be on understanding the experiences of specific, isolated groups and within that context of dementia, rather than placing these into the wider field of knowledge about inequalities. This has resulted in it being more difficult to see the broader picture of inequalities in dementia. This, as we noted in the introduction, has not helped develop policy clearly focused on addressing inequalities. The resultant restricting of perspective does not facilitate looking across groups at what may be the systemic issues causing inequalities in dementia care, such as biases and assumptions in diagnostic processes, understanding of dementia, and carer arrangements, as well as stigmatising attitudes and behaviours inbuilt in services. Such issues were evident across the domains we explored above and were not unique to any one group of people, even though their detailed manifestations may be specific to them.

By not connecting with the wider evidence and debates concerning inequalities, dementia risks reducing consideration of unfair problems in care to issues in individuals/communities and to isolated barriers, rather than connecting with debates about the more systemic interplay of factors causing inequalities (e.g. [93]). Adopting a lens of inequality would enable dementia research to draw on wider bodies of knowledge concerning inequalities and their causal mechanisms, e.g. the social determinants [1], inequalities in long-term conditions generally [94], and how such structural issues interplay with individual circumstances and agency e.g. [95, 96]. What can we learn from this inequalities evidence for understanding improving diagnosis processes and post-diagnostic services for people experiencing poorer ones at the moment? We may understand, for example, that people can have different levels/forms of health literacy regarding dementia, but see that this understanding is structured by social circumstances but not determined by them and that agency may be altered by changing social contexts. This does not locate blame or responsibility in individuals but allows working with their sense of control with understanding how circumstances shape their agency. Nor, of course, does this assume that everyone with a characteristic has the same expectations and circumstances.

Chen et al. [97] have raised concerns that the incidence of dementia may not be declining as previously thought, and that there may be widening inequalities of incidence in England caused by increasing health risk factors in socially disadvantaged populations. This reinforces the need to understand dementia in a social context and in relation to other social and health inequalities.

Our second overarching theme from this mapping, and related to the points just made, is that *little of the research we found considers an intersectional perspective on people’s characteristics, circumstances and experiences* when seeking support for dementia. Intersectionality moves consideration away from thinking about people’s lives in terms of one characteristic or axis of their identity, such as gender or race, to consider the multiple and simultaneous interactions in a person’s life and the impact of these on their identities and lives [98–100]. In so doing, it draws attention to the danger of essentializing categories and treating populations with a shared characteristic as homogenous, such as the often-implicit assumption that all women are the same and have a shared set of experiences and outlooks (e.g., white, middle class, heterosexual and able-bodied).

Further, an intersectional perspective steers away from thinking in simple additive terms of the impact of people’s characteristics to understanding their interacting and potentially compound effects on people’s lives. For example, relationships between ethnicity and inequalities across health are complex [5], reflecting, among other things, different patterns of migration, but also multiple interactions with other characteristics, such as gender and socioeconomic circumstances [101]. Intersectionality is increasingly articulated and applied as a perspective to understand health inequalities and how to address them (e.g., [102–105]). Our call for an intersectional understanding of inequalities in dementia resonates with Giebel’s [6] call and the development of a dementia inequalities framework drawing in factors at the levels of the individual, community, and society.

As we have noted, some authors examining inequalities in dementia care have identified interactions between people’s circumstances and different characteristics such as ethnicity and socioeconomic status. Insightful examples of work in this area include that exploring the intersections of racism, dementia and stigma (e.g. [106]), and sexuality, ableism, ageism and dementia [107, 108] show the cumulative impacts of disadvantages. However, in the main, literature on inequalities in dementia care tends to focus on a single characteristic, such as ethnicity, implying this to be the sole determining factor in experience of dementia diagnosis and care. We would urge a more complex, intertwined and contextualised analysis. In the context of Wolverhampton, for example, communities from the same Indian heritage can have quite different socioeconomic backgrounds and migration experiences (e.g. [109]), which may mean that an intervention aimed at improving dementia care for a presumed uniform Indian population does not work for everyone sharing that characteristic.

Another example of an intersectional understanding would be not assuming that all women had the same experiences of dementia or of being a carer. Rather, we would need to examine how being a woman intersected with other aspects of identity, such as ethnicity and socioeconomic status, to shape experiences and expectations. Failure to do this might mean that we organise services for women from particular backgrounds (e.g. white, heterosexual) and, hence, exclude others who do not share this exact same intersectional web of experiences and characteristics.

Our third and final overarching theme from mapping this terrain is that *of finding solutions to inequalities in dementia care*. The strongest evidence we found relates to describing the inequalities and barriers people face in accessing diagnosis and services. Robust evidence for specific policies and interventions to address the inequalities is far sparser. Addressing this gap in our evidence should be an urgent priority if individuals and communities are not to be left behind in dementia support. This interacts with our second theme – the need for a sophisticated understanding of the intersectionality of inequalities and, hence, recognising that solutions need to consider multiple aspects of people’s lives if they are to work to improve unfair care systems.

From this analysis of the broad contours of evidence concerning inequalities and dementia care, we recommend:

i) Dementia research would benefit from a more explicit focus on inequalities and inequities to allow drawing together of evidence across groups of people.
ii) Research in dementia should build a stronger dialogue with evidence concerning inequalities and inequities beyond dementia care, particularly in analysing underpinning causal mechanisms for inequalities and related solutions.
iii) More, and more robust, evidence is needed on the intersection of people’s characteristics and inequalities in dementia care.
iv) Stronger evidence is needed on interventions to reduce inequalities and improve access to care and achievement of better outcomes.
v) To help bring points above together, we need high-quality research on the expectations and agency of people regarding dementia and care systems. Understanding these, with an intersectional view on inequality, would enable us to better target resources to the levels and forms of support that people want to achieve equity.
vi) National policy frameworks bearing on dementia should more explicitly adopt an inequalities perspective to address systemic issues in care systems.
vii) Local care systems should employ an intersectional perspective on improving dementia care rather than assuming people all want similar things, even those sharing a particular characteristic.

## Conclusion

In England the landscape within which dementia support is planned and delivered continues to change. This includes continuing delays and uncertainty regarding social care reform, resource pressures across health and social care (notably workforce) and reshaping the governance arrangements aimed at greater integration generally within health care and with social care (i.e. Integrated Care Systems). It would be timely to consider improvements to dementia care and support within this developing landscape from the perspective of inequalities to ensure that care systems develop as rapidly as possible to overcome these inequalities. An inequalities lens and a broader understanding of the evidence on inequalities would help to identify structural barriers that people face to accessing dementia diagnoses and support. Taking this further, considering the intersectionality of people’s characteristics and experiences and the barriers to support would help in achieving the goal of delivering high-quality personalised support to all.

## Data Availability

No datasets were generated or analysed during the current study.
